# [Corrigendum] CDKN3 is an independent prognostic factor and promotes ovarian carcinoma cell proliferation in ovarian cancer

**DOI:** 10.3892/or.2026.9218

**Published:** 2026-11-02

**Authors:** Tianren Li, Hui Xue, Yi Guo, Kejun Guo

Oncol Rep 31: 1825-1831, 2014; DOI: 10.3892/or.2014.3045

Subsequent to the publication of the above paper and an Expression of concern statement (doi. 10.3892/or.2025.9008) that was published after a concerned reader drew the Editor's attention to the fact that the cell invasion assay data shown in the three panels in Fig. 6A on p. 1830 appeared to contain overlapping sections at different orientations (all three panels), the authors have now replied to the Editorial Office. After carefully reviewing their original image files, the authors have realized that the photographs were inadvertently mistakenly selected during the manuscript preparation and figure editing stage. Subsequently, they have selected replacement representative images for the data panels in Fig. 6A in this figure, verified the experimental data, and confirmed that the quantitative analyses and conclusions reported in the manuscript remain unchanged.

The revised version of Fig. 6, now showing data from one of the repeated experiments in Fig. 6A, is shown below. Note that the revisions made to this figure do not affect the overall conclusions reported in the paper. The authors are grateful to the Editor of *Oncology Reports* for allowing them the opportunity to publish this Corrigendum, and apologize to the readership for any inconvenience caused.

## Figures and Tables

**Figure 6.**
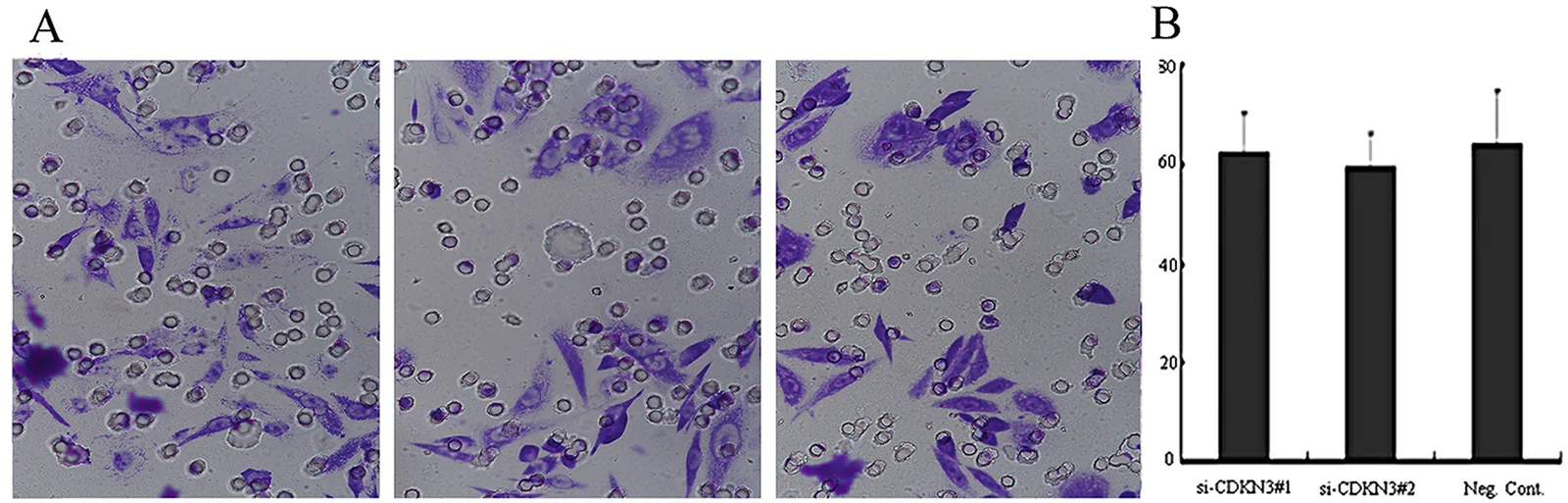
(A) CDKN3 siRNA treatment did not have a significant blocking effect on cell invasion in the OVCAR3 cell line. (B) The number of invasive cells was counted and no difference was observed among the cell graph.

